# Developments in Bio-Inspired Nanomaterials for Therapeutic Delivery to Treat Hearing Loss

**DOI:** 10.3389/fncel.2019.00493

**Published:** 2019-11-06

**Authors:** Christopher Rathnam, Sy-Tsong Dean Chueng, Yu-Lan Mary Ying, Ki-Bum Lee, Kelvin Kwan

**Affiliations:** ^1^Department of Chemistry and Chemical Biology, Rutgers, The State University of New Jersey, Piscataway, NJ, United States; ^2^Department of Otolaryngology Head and Neck Surgery, Rutgers New Jersey Medical School, Newark, NJ, United States; ^3^Stem Cell Research Center and Keck Center for Collaborative Neuroscience, Rutgers, The State University of New Jersey, Piscataway, NJ, United States; ^4^Department of Cell Biology and Neuroscience, Rutgers, The State University of New Jersey, Piscataway, NJ, United States

**Keywords:** inner ear, nanoparticle, hydrogel, drug delivery, hearing loss

## Abstract

Sensorineural hearing loss affects millions of people worldwide and is a growing concern in the aging population. Treatment using aminoglycoside antibiotics for infection and exposure to loud sounds contribute to the degeneration of cochlear hair cells and spiral ganglion neurons. Cell loss impacts cochlear function and causes hearing loss in ∼ 15% of adult Americans (∼36 million). The number of individuals with hearing loss will likely grow with increasing lifespans. Current prosthesis such as hearing aids and cochlear implants can ameliorate hearing loss. However, hearing aids are ineffective if hair cells or spiral ganglion neurons are severely damaged, and cochlear implants are ineffective without properly functioning spiral ganglion neurons. As such, strategies that alleviate hearing loss by preventing degeneration or promoting cell replacement are urgently needed. Despite showing great promise from *in vitro* studies, the complexity and delicate nature of the inner ear poses a huge challenge for delivering therapeutics. To mitigate risks and complications associated with surgery, new technologies and methodologies have emerged for efficient delivery of therapeutics. We will focus on biomaterials that allow controlled and local drug delivery into the inner ear. The rapid development of microsurgical techniques in conjunction with novel bio- and nanomaterials for sustained drug delivery appears bright for hearing loss treatment.

## Introduction

### Drugs for Treating Inner Ear Disease

Sudden sensorineural hearing loss (SSHL), Meniere’s disease (MD), and autoimmune inner ear disease are prevalent inner ear disorders seen in the clinic. Sudden sensorineural hearing loss affects 1–6 people per 5,000 every year (National Institute on Deafness and Other Communication Disorders) and is characterized as an idiopathic hearing loss that occurs suddenly or rapidly over a few days. Often, SSHL is associated with tinnitus, a ringing, or other auditory perception in the absence of an external stimulus (Schreiber et al., 2010). Meniere’s disease is a disorder of the inner ear with unknown etiology. The symptoms of MD include episodes of spontaneous, recurrent vertigo accompanied by fluctuating hearing loss, intermittent tinnitus and characterized by endolymphatic hydrops (Havia et al., 2002; Nakashima et al., 2016). Autoimmune ear diseases (AIED) are characterized by the bilateral progressive sensorineural hearing loss that occurs over the course of weeks to months, with patients responding to the administration of immunosuppressants. Primary AIED has restricted pathology to the ear but may involve secondary autoimmunity. Although the etiology of the disorder is likely immune-mediated, there is no direct evidence that autoimmunity is the underlying cause of the disease. Autoimmune inner ear diseases include Cogan syndrome, Wegener granulomatosis, systemic lupus erythematosus, and various vasculitis (Ruckenstein, 2004).

In the clinic, patients diagnosed with SSHL or MD are treated with steroids, aminoglycosides, antioxidants (L-N-acetylcysteine), apoptosis inhibitors, or *N*-methyl-D-aspartate (NMDA) inhibitors. For AIED, cytotoxic agent cyclophosphamide is used. To date, only steroids and aminoglycoside are clinically available therapies for intratympanic administration to the inner ear. Intratympanic steroid injection is the most prevalent clinical application to restore acute hearing loss in all inner ear diseases. While the specific action of the steroids in the hearing apparatus is uncertain, steroid treatment likely improves hearing by reducing inflammation and swelling in the hearing organs. Use of intratympanic dexamethasone injections to treat sudden hearing loss may also attenuate symptoms of tinnitus (Garduno-Anaya et al., 2005). A number of clinical studies have been published on intratympanic injection of steroids for the treatment of acute hearing loss and vertigo exacerbation in MD, in addition to systemic therapy (Schoo et al., 2017). Furthermore, intratympanic administration of aminoglycosides (i.e., gentamicin) to chemically ablate vestibular hair cells is now an alternative and less invasive procedure for achieving chemical labyrinthectomy for uncontrolled vertigo spells in MD while preserving auditory function (Hill et al., 2006).

## Current Strategies for Inner Ear Drug Delivery

The current treatment standard for inner ear disorders is the intratympanic injection of liquid formulations of drugs. While intratympanic injection is the most common method for delivery of drugs to the inner ear, other methods such as intracochlear delivery are also available for extreme cases. In either case, several barriers need to be overcome for efficient drug delivery.

### Intratympanic Drug Delivery

The tympanic membrane (TM) is a thin, cone-shaped membrane that separates the external ear from the middle ear. The TM has low permeability to most substances and is considered an impenetrable barrier for drug delivery to the inner ear. For intratympanic drug delivery, the integrity of TM is compromised before drugs are injected into the middle ear cavity where diffusion into the inner ear occurs. The shape of the external canal and differences in cone angle and depth of TM in humans hinder direct and precise administration of drugs (Volandri et al., 2011). Successful employment of steroids by intratympanic delivery for inner ear disease treatment was first reported about three decades ago (Itoh and Sakata, 1991). Since then, intratympanic injections are the preferred delivery method for treatment of SSHL, MD or vertigo because local delivery avoids the many side effects associated with systemic drug delivery (Chandrasekhar, 2001; Doyle et al., 2004). To date, delivery of either steroids or aminoglycosides is done by intratympanic injection to avoid the bony structure of the otic capsule and the TM. Delivery of small molecules or biologics in the inner ear by intratympanic injections still need to overcome several physical and cellular barriers (Chandrasekhar, 2001; Doyle et al., 2004). Physical barriers such as the RWM and other membranous partitions prevent drugs from diffusing from the middle ear cavity where the therapeutics reside.

The majority of small molecules delivered intratympanically likely enter through the RWM by passive diffusion into the inner ear. Diffusion of small molecules after intratympanic delivery results in large gradients along the length of the cochlea (Salt and Plontke, 2009). Extraneous obstructions due to anatomic variations in the round window niche contribute to the wide variability in delivery and dosage of drugs thus limiting reproducible clinical efficacy (Alzamil and Linthicum, 2000). Intratympanic injections are performed either without assisted visualization or with a micro-endoscope to assist in removing potential round window niche obstructions (Plontke et al., 2002). Once injected, the liquids in the middle ear cavity will eventually drain into the Eustachian tube that connects the middle ear cavity into the nasopharynx. The TM heals after the surgical procedure while the efficacy of treatment is monitored. The inconsistent nature of intratympanic injections due to anatomical variations, variability in treatment protocols and the different biological effects of various inner ear cell types influence treatment efficacy.

### Drug Entry Through the Round and Oval Windows

For intratympanic administration, drugs in the middle ear cavity can diffuse into the basal cochlear turn through the RWM, and possibly through the oval window. The RWM is a semi-permeable membrane separating the middle ear from the inner ear. It is composed of three layers: an outer epithelial layer, a middle connective layer, and an inner cellular layer facing the scala tympani perilymph of the cochlea. RWM permeability is affected by many factors in normal and pathological conditions (Goycoolea, 2001). The varied shapes of the ear canal in humans often introduce difficulty in visualizing RWM during intratympanic administration and the obstruction of RWM by plugs of connective or fibrosis tissue can lead to interpatient variability in intratympanic administration (Salt and Plontke, 2005).

**FIGURE 1:**
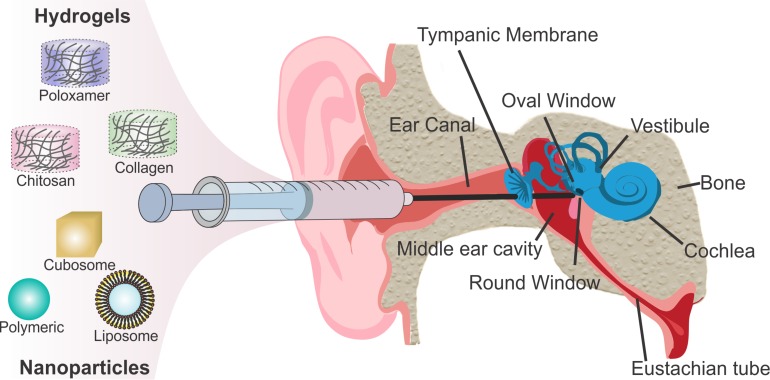
Scheme of intratympanic injection of biomaterials to the round window for inner ear therapy.

The oval window allows access to the scala vestibule of the inner ear from the middle ear cavity. The oval window is covered by the footplate of the stapes and is attached by the annular ligament. Experimental results suggest that the oval window is more permeable to compounds than RWM, likely due to the cellular composition of the structure. Delivery through the oval window may be useful in treating vestibular dysfunction and provides a promising route of drug delivery to the vestibular system, which houses the saccule, utricle, and semi-circular canals (Figure 1; King et al., 2011, 2013, 2017).

### Intracochlear Drug Administration

To overcome drug loss from the middle ear space, intracochlear administration allows direct drug entry into the cochlea. To this end, several methods have been developed to provide direct access to the cochlea, including canalostomy followed by direct injection, osmotic pumps, or cochlear implant mediated delivery (Ayoob and Borenstein, 2015). Achieving even distribution of therapeutics throughout the cochlear turns may be difficult. This is due to the varying diameters of the coiled cochlea, slow diffusion rate from base to apex of the cochlea, poor control of targeted delivery to the cells in different cochlear turns and delayed intracochlear fibrosis that develops over time. Direct access to the cochlea is generally avoided because it requires surgical and destructive techniques that can lead to co-morbidities and exacerbate inner ear damages (Borenstein, 2011). Clinicians typically employ intracochlear delivery only as the last option or if they are already performing surgery for other ailments.

## Barriers to Inner Ear Drug Delivery

The bony otic capsule housing the cochlea and vestibular organs is the major anatomic and physiological barrier to inner ear drug delivery. It is both the densest bone and most inaccessible location in the human body for surgical procedures. To avoid the otic capsule, systemic drug delivery allows drugs to enter the inner ear intravascularly through the blood vessels. The intravascular route is an attractive, non-invasive strategy to avoid the inaccessible anatomy. The drugs likely travel through the labyrinthine artery, which branches into the spiral modiolar artery, vestibulocochlear artery, and the anterior vestibular artery. The spiral modiolar artery supplies blood into the stria vascularis and organ of Corti and allows drugs to enter into these regions. The permeability of drug through the blood-labyrinth barrier (BLB) at the luminal surface of inner ear capillaries restricts the entry of many blood-borne compounds into inner ear tissues. Similar to the blood-brain-barrier, the BLB is characterized by endothelial cells that seal the luminal surface of the inner ear capillaries using tight and adherent junctions. The BLB preferentially excludes high molecular weight compounds and decreases their entry into the inner ear. Local administration avoids BLB restricted systemic delivery into the inner ear.

### Drug Clearance From the Middle Ear Cavity

In the middle ear cavity, the round and oval window membranes are potential physical barriers that prevent drug permeation and accumulation in the cochlea. In intratympanic delivery, while drugs reside in the middle ear cavity, the liquid drains from the Eustachian tube, a canal that connects the middle ear cavity to the throat and the back of the nasal cavity. Drainage is facilitated by mucociliary flow, causing drug loss and short drug residence time in the middle ear space. Increasing drug residence in the middle ear cavity allows for sustained drug delivery and limits drug clearance by drainage through the Eustachian canal or excretion out of the inner ear BLB (Salt, 2005; Salt and Plontke, 2005). Effective drug clearance in the middle ear causes short drug residence time and decreases drug concentrations after administration resulting in less effective treatment.

### Drug Clearance From the Cochlea

Once cochlear delivery is achieved, the residence of therapeutics in the cochlea affects the efficacy of treatment. A cross-section of the cochlea reveals three separate compartments: the scala vestibuli, scala media, and scala tympani. Tight cellular barriers separate the Na^+^ rich perilymph in the scala media and scala vestibule from the K^+^ rich endolymph in the scala media. These barriers vary in the degree of permeability, which complicates the assessment of pharmacokinetic spread and elimination of drugs. Fluid exchange and maintenance of salt concentrations in the cochlea alter residence time and drug concentrations in the cochlea duct (Salt and Plontke, 2005). Unlike other bodily fluids, cochlear fluid composition, cochlear fluid homeostasis, and generation and regulation of the endocochlear potential by K^+^ secretion contributes to the distribution of drugs throughout the cochlea (Wangemann, 2006) and results in highly variable or unpredictable pharmacokinetics.

A potential route that decreases the residence time of therapeutics in the inner ear is drainage through the cochlear aqueduct, which connects the perilymphatic space of the cochlear basal turn to the subarachnoid space of the posterior cranial fossa. The cochlear aqueduct maintains fluid and pressure balance between the inner ear perilymph and cerebral spinal fluid (CSF) (Gopen et al., 1997). Perilymph sampling from the basal turn of scala tympani for pharmacokinetic studies of inner ear drug delivery systems is contaminated by CSF (Salt and Hirose, 2018). CSF dilutes and hinders effective concentration of drugs after intratympanic or intracochlear administration at the cochlear base. Understanding the microanatomy, fluid flow, and characteristics of the different cell types in the inner ear are important to achieve a uniform and sustained delivery of drugs or therapeutics in the cochlea.

## Biomaterials for Local Drug Delivery

The fragile nature of the inner ear, the numerous membranous structures and the complex biological barriers designed to protect the inner ear from harmful exogenous materials make delivery to the inner ear extremely challenging. Developing non-ototoxic biomaterials that can bypass the tissue-specific biological barriers are required for efficient delivery. Systemically administered drug or biologic must cross the blood labyrinth barrier to reach the inner ear (Juhn et al., 1982). Usually, only a small amount of the therapeutic reaches the inner ear. Therefore, high doses of therapeutics are required to achieve effective concentrations for efficacious treatments. Systemic administration of high drug concentrations, particularly steroids, has numerous side effects. To avoid such side effects, intratympanic injection of therapeutics to the middle ear cavity allows local organ targeted delivery.

Even though intratympanic injection allows for local drug delivery, it results in variable drug kinetics and lack of cellular specificity. Newer approaches for drug delivery into inner ear have considered these limitations and employed novel biomaterials to sustain prolonged drug exposure to targeted cells with minimal systemic side effects. Such delivery methods will allow for a single local administration without repeated dosing in an office-based procedure. Development of biomaterials that overcome cellular toxicity, control degradation kinetics and modulate drug distribution after delivery need to be addressed.

To sustain delivery, limit degradation and allow even distribution in the inner ear, two major classes of biomaterials have been developed: hydrogels and nanoparticles. Soluble drugs can be loaded into hydrogels placed at the RWM for controlled and sustained drug diffusion from the middle ear space. Coupling drugs to nanoparticles impede drug degradation and can be tuned for fast diffusion and cell-specific targeting in the inner ear. When designing such biomaterials, additional considerations include efficacy, toxicity, and long-term degradation. Delivery of biomaterials is well studied in a variety of organs such as the eye, bones, heart. Requirements for each targeted organ entails a unique design and modifications to safely and effectively function as therapeutic carriers (Langer and Peppas, 1981; Panyam and Labhasetwar, 2003; Couvreur, 2013; Li and Mooney, 2016). To this end, recent advances in material science and nanochemistry have led to the rapid development of biomaterial systems for inner ear drug delivery (Table 1).

**TABLE 1:** Common biomaterial used for drug delivery to the inner ear.

**Material**	**Structure**	**Key advantage**	**Method of drug loading**	**References**
Poloxamers	Hydrogel/Nanoparticles	Thermogelation, tunable drug release, biodegradable, chemically defined, porosity	Encapsulation, chemical linking	Dumortier et al., 2006; Wang et al., 2009, 2011; Salt et al., 2011; Engleder et al., 2014; Honeder et al., 2014;
Chitosan	Hydrogel/Nanoparticles	Cationic, mucoadhesive, biocompatible, biodegradable, porosity	Electrostatic, encapsulation, chemical linking	Dai et al., 2018; Kayyali et al., 2018
Collagen/Gelatin	Hydrogel/Nanoparticles	Naturally found in humans, biocompatible, biodegradable gelatin-low immunogenicity	Encapsulation, chemical linking	Endo et al., 2005; Iwai et al., 2006; Lee et al., 2007; Inaoka et al., 2009; Kikkawa et al., 2009; Van Vlierberghe et al., 2011; Lambert et al., 2016
PLGA	Hydrogel/Nanoparticles	Biodegradable, tunable size, tunable degradation, chemically defined, FDA approved, porosity	Encapsulation, tethering	Tamura et al., 2005; Zou et al., 2010b; Cai et al., 2014, 2017; Dai et al., 2018
SPION	Nanoparticles	T2 MRI contrast agent, magnetofection, FDA approved for imaging	Adsorption, Tethering	Thaler et al., 2011; Ramaswamy et al., 2017; Zou et al., 2017
Polymersomes	Nanoparticles	Ease of functionalization, biocompatible, versatile drug loading, chemically synthesized	Encapsulation, tethering	Meng et al., 2009; Roy et al., 2010; Zhang et al., 2011a, 2012; Surovtseva et al., 2012
BSA	Nanoparticles	Biocompatible, biodegradable	Encapsulation	Yu et al., 2014
Lipid nanocapsules	Nanoparticles	Small size, physical stability, ease of manufacture, high hydrophobic drug loading, chemically and structurally defined	Encapsulation	Zou et al., 2008; Zhang et al., 2011b
Liposomes	Nanoparticles	Biocompatible, FDA approved, hydrophobic and hydrophilic drugs	Encapsulation, tethering	Fröhlich, 2012; Kayyali et al., 2018; Yang et al., 2018
Cubosomes	Nanoparticles	Biodegradable, loads all drug types, high drug loading, physical stability, chemically and structurally defined	Encapsulation	Azmi et al., 2015; Bu et al., 2015
Silver nanoparticles	Nanoparticles	Anti-fungal, Anti-bacterial, ease of synthesis, chemically and structurally defined	Tethering, adsorption	Zou et al., 2015
Silica	Nanoparticles	Biodegradable, ease of surface tuning/modification, porosity, chemically and structurally defined	Adsorption, tethering	Praetorius et al., 2007; Wise et al., 2016; Xu et al., 2018

### Hydrogels for Drug Retention and Controlled Release of Therapeutics

**FIGURE 2:**
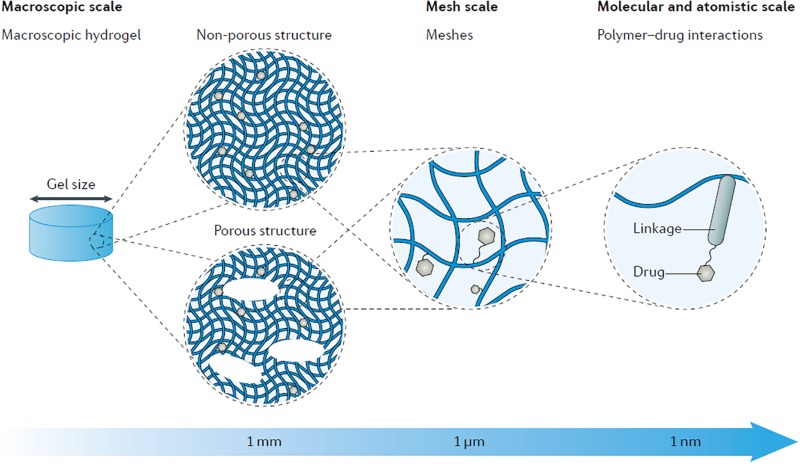
Schematic showing the multi-scale tunability of hydrogels for drug delivery applications, showcasing the versatility of drug delivery properties based on material characteristics. By modulating material properties such as composition, porosity, and intramolecular bonding the degradation and drug release kinetics can be tuned as well as other properties such as gelation temperature and shear thinning mechanics. Reprinted by permission from Springer Nature: Nature Reviews Materials, Designing hydrogels for controlled drug delivery, Jianyu Li, David J. Mooney, Copyright 2016.

A major hurdle of intratympanic or intracochlear delivery is controlling residence time and sustained release to achieve therapeutic drug concentrations in the inner ear. Hydrogels helps retain drugs for sustained release. Hydrogels are hydrophilic polymeric networks that retain water and swell with a very large water fraction. Hydrogels are of particular interest for drug delivery because of their chemical functionality, biocompatibility, tunable physical properties, drug loading capability, and degradation capability (Peppas and Sahlin, 1996; Hoare and Kohane, 2008). Hydrogel-based drug delivery systems into the inner ear involve introducing a liquid that gels in the middle ear cavity near the RWM. Retaining the drug in the solidified hydrogel allows the drug to persist in a local region, prolong diffusion across the RWM and release of drugs at therapeutically meaningful concentrations (El Kechai et al., 2015). Viscosity of hydrogels increases the retention time of drugs, improves drug kinetics by increasing residence time and allows equilibrium to be reached for even distribution. Hydrogels vary in their molecular interactions, ionic charge, physical appearance, and chemical composition such as the source of the monomer (Figure 2; Li and Mooney, 2016), but can be classified into natural and synthetic products. Natural products are some of the best-studied hydrogels due to biocompatibility as observed by the lack of cellular toxicity and negligible immune response after introducing the biomaterial into the human body. Synthetic hydrogels are becoming more prevalent due to increased control over chemical functionality, mechanical properties, drug loading, and degradation kinetics (Ahmed, 2015).

### Synthetic Poloxamer Hydrogels

Poloxamers are synthetic polymers made of a central block of poly (propylene oxide) (PPO) and two flanking blocks of poly (ethylene glycol) (PEG). Poloxamers are extensively developed for drug delivery because their gel transition temperature can be adjusted by varying the concentration of poloxamer. The poloxamer can form a thermo-responsive gel that is a liquid at room temperature for handling ease, resuspension, and injection of a drug, while triggered to gel at body temperature *in situ* when applied to the RWM. Of the many poloxamers studied, poloxamer 407, where the 40 represents the PPO molecular mass (4,000 g/mol), and the 7 represents the PEG content (70%), is the main synthetic hydrogel used for inner ear delivery. This poloxamer has been used to deliver a variety of different drug types into the inner ear (Dumortier et al., 2006). Compared to intratympanic injection of dexamethasone, poloxamer 407 loaded with micronized dexamethasone (mDex) delivered to the guinea pig round window provided sustained release of the drug, increased the overall concentration in the perilymph by ∼1.6-fold and increased the residence time of the drug by ∼24 fold. Injected dexamethasone solutions show an initial spike in concentration before clearing from the perilymph within 12 h, while the mDex hydrogel formulations result in 10 days of sustained release. Use of poloxamer 407 highlights its suitability for long term drug delivery (Wang et al., 2009). Prolonged-release of dexamethasone would be more beneficial for the treatment of inner ear disease rather than a single bolus dose that is rapidly cleared. The study also demonstrates that mDex delivery using poloxamer 407 led to more homogenous distribution of dexamethasone along the length of the cochlea (Salt et al., 2011).

Another advantage of using poloxamers is the ability to deliver low solubility therapeutics. Formation of micelles in the hydrogel facilitates loading of hydrophobic drugs and gel formation (Engleder et al., 2014). Low solubility drugs such as dexamethasone and methylprednisolone are more easily incorporated in the poloxamer hydrogel compared to aqueous solution (Wang et al., 2011). Triamcinolone Acetonide (TAAc), a glucocorticoid for treatment of inner ear disease, was loaded into poloxamer 407 hydrogels. Intratympanic injection of the TAAc loaded hydrogels near the round window allowed for 10 days of sustained drug release at high levels. Levels of TAAc were significantly higher (up to 600 fold) in the perilymph than in the plasma, suggesting local release of drug through the RWM with minimal release into the circulatory system. Hydrogel treatment, however, showed temporary ABR threshold shifts which returned back to normal 10 days without histological damage or reduction in hair cell numbers. Intratympanic injection of the hydrogel into the middle ear likely prevents movement of the middle ear bones and perturbs sound transmission to the inner ear. Degradation of the hydrogel reverses the temporary ABR threshold shift (Honeder et al., 2014). These examples demonstrate sustained drug delivery through the RWM using poloxamers.

### Natural Chitosan Hydrogels

Natural polymer hydrogels are based on collagen, hyaluronic acid, and chitosan. The main advantage of using these polymers are biocompatibility, making them excellent non-toxic drug delivery systems. Chitosan has been developed for many years due to its biocompatibility and biodegradability. Chitosan is found in the outer skeleton of shellfish and has cationic characteristics that confer mucoadhesive property which allows it to adhere to the outer epithelium of the RWM by electrostatic interactions (Sogias et al., 2008). The selective attachment of chitosan allows for specific placement at the RWM. Chitosan glycerophosphate (CGP) has also been used to make hydrogels due to its favorable thermoresponsive properties to gel at body temperature, its mucoadhesive properties, and its ability to harbor a variety of payloads. Delivery of dexamethasone into the murine inner ear using CGP hydrogels demonstrate they adhere to the round window niche and shows release of a high percentage (92%) of the dexamethasone in the perilymph after 5 days (Paulson et al., 2008).

In addition to corticosteroids, CGP hydrogels have been used to treat MD by delivering the ototoxic drug gentamicin to chemically ablate vestibular hair cells. By intratympanic injection, gentamicin enters both the vestibule and the cochlea but selectively accumulates in vestibular hair cells allowing for preferential vestibular hair cell ablation (Lyford-Pike et al., 2007). CGP hydrogel delivery allowed sustained gentamicin release over 7 days compared to an injection of liquid gentamicin which declined after 1 day (Xu et al., 2010). Despite sustained release, a gentamicin concentration gradient developed in the cochlea from the base-to-apex, exposing gentamicin to the basal portion of the cochlea at higher doses and for a longer period (Luo and Xu, 2012). The side effect of increased gentamicin at the base of the cochlea may lead to high-frequency hearing loss.

To gain more control over the timed-release of drugs, the addition of chitosanase, an enzyme that degrades chitosan hydrogel can be used to “turn off” drug delivery. Treatment with chitosanase quickly degrades the CGP-hydrogel, which is then cleared through the Eustachian tube. This results in burst release and accumulation of low drug concentrations in the perilymph. The “turn off” system for CGP-based hydrogel drug delivery can be employed to quickly terminate drug delivery and avoid unwanted side-effects from sustained or prolonged drug exposure (Lajud et al., 2013). Chitosan gels can be used to deliver many different drugs, but the intended use and effects of the drugs for inner ear treatment can be tuned for slow sustained delivery or controlled burst release. These studies demonstrate the flexibility for chitosan-based hydrogels as a controllable local drug delivery system to the inner ear (Lajud et al., 2015).

### Natural Collagen or Gelatin Hydrogels

Another natural polymer commonly used for inner ear delivery is collagen or its denatured form gelatin. Both are found in the human body and are biocompatible. When combined with various types of cross-linkers, collagen and gelatin can form hydrogels for controlled drug release (Van Vlierberghe et al., 2011). Delivery of biologics, such as growth factors that promote inner ear cell survival, are limited by the short half-life of these proteins. Use of the biocompatible natural hydrogels ensure that the proteins retain biological activity, reduce degradation and allow for prolonged release.

Gelatin hydrogel delivery of insulin-like growth factor 1 (IGF1) to the inner ear after noise-induced hearing loss in guinea pigs increased outer hair cell survival. The positively charged recombinant human IGF1 protein electrostatically interacts with negatively charged gelatin hydrogel. IGF1 release over 7 days reduced auditory brainstem recording (ABR) thresholds and prevented hearing loss (Iwai et al., 2006; Lee et al., 2007). Exogenous hepatocyte growth factor (HGF) has been shown to be otoprotective and promote hair cell survival after toxic insults (Kikkawa et al., 2009). HGF soaked gelatin hydrogel administered to the round window of a noise-damaged guinea pig prevented ABR threshold increase and outer hair cell loss in the basal region of the cochlea (Inaoka et al., 2009). Brain-derived neurotrophic factor (BDNF), a factor that increases spiral ganglion neuron survival after ototoxic damage, was delivered to the RWM by gelatin hydrogel. Sustained delivery by encapsulating BDNF in the gelatin hydrogel increased BDNF concentration in the perilymph over 100 fold, which was detected even after 7 days. Sustained exposure to BDNF prevented elevated ABR thresholds and preserved spiral ganglion neurons from ototoxic insult (Endo et al., 2005).

Use of hydrogels for slow release of drugs and biologics into the inner ear can be important for long-term application. Hydrogels are the lowest hanging fruit in terms of biomaterials for delivery to the inner ear because of their non-toxic and biologically safe nature of the material; however, safety and efficacy for the treatment of inner ear disorders in longitudinal studies still need to be validated. In fact, several companies are performing clinical trials for gel-based formulations for the treatment of inner ear disorders (Lambert et al., 2016). While hydrogels have the distinct advantage of increasing retention time and providing sustained drug release at the round window, they do not address the challenges of slowing drug degradation and ensuring cell-specific delivery. Preventing drug degradation allows prolonged residence of effective concentration drugs at the delivery site, while cell-specific delivery increases therapeutic efficacy and limits deleterious side effects.

### Nanoparticles for Improved Diffusion and Clearance Prevention

**FIGURE 3:**
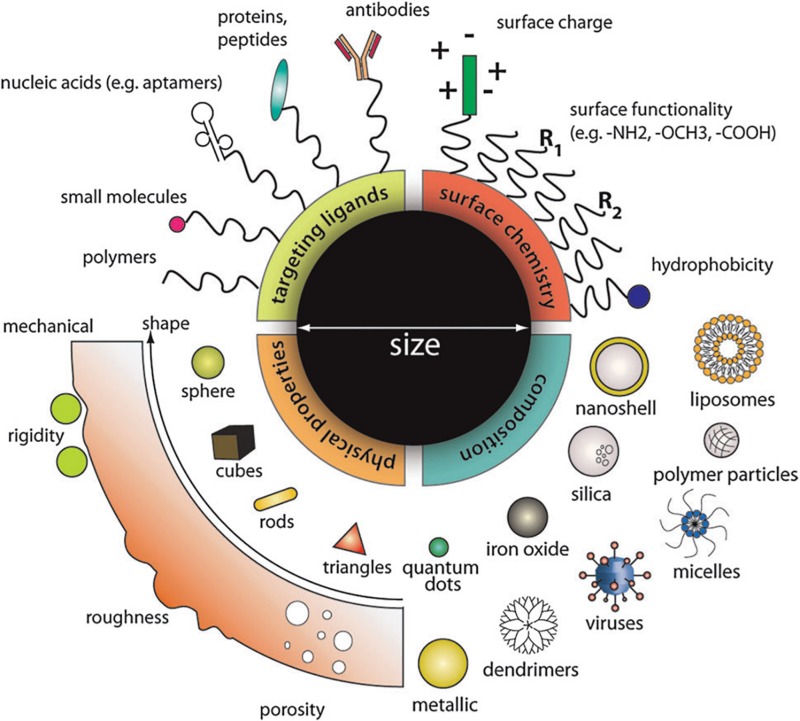
Material composition, surface properties, and functional groups can all be tuned to modulate the functional properties of nanoparticles such as drug loading and release kinetics, fluorescence, cell targeting, diffusion, cellular uptake, and many more. The modular nature of nanoparticles makes them ideal for optimizing the delivery of molecules for specific bioapplications. Reprinted with permission from Ref 55. Copyright 2011 Royal Society of Chemistry.

The design of nanoparticles is applicable for delivering a wide variety of therapeutics in different diseases. Coupling therapeutics to nanoparticles can alter drug degradation and diffusion kinetics through tissue barriers. Nanoparticles prevent drug degradation by providing a protective barrier around the molecule. Diffusion kinetics of drugs can be altered by modifying nanoparticle surface properties, such as charge and hydrophobicity to increase drug circulation time, tissue penetration and cellular uptake. Nanotechnology allows tuning of designer nanoparticles properties to increase drug accumulation and stability, as well as provide cell-specific delivery of therapeutics to the tissue of interest (Figure 3; Chou et al., 2011). In addition, nanoparticle platforms allow multiple drugs to be loaded in order to increase drug delivery efficiency and efficacy. Nanoparticle-based drug delivery into the inner ear remains relatively unexplored.

### Polymeric Nanoparticles

Polymeric nanoparticles are advantageous for drug delivery due to their biocompatibility, small size, degradation tunability, and relatively simple synthesis procedure. Because of this, polymeric nanoparticles have been used in a wide range of drug delivery applications including cancer therapies, diagnostics, molecular imaging, and a myriad of other applications (De Jong and Borm, 2008; Janib et al., 2010). PLGA is one of the most well studied polymeric nanoparticles due to its biocompatibility, ease of surface modification, biodegradability, and ability to encapsulate drugs. Early studies utilizing a rhodamine-conjugated poly (lactic-co-glycolic acid) (PLGA) nanoparticles were detected by fluorescence microscopy. Systematic delivery of rhodamine-conjugated PLGA nanoparticles accumulated in the liver, kidney, and cochlea of guinea pigs. Long-term residence of the nanoparticles was observed only in the liver but not in the cochlea or kidney. Limited accumulation was likely due to tissue-specific barriers, increased clearance or rapid degradation of particles. To enhance local concentrations in the cochlea, PLGA nanoparticles were suspended in gelfoam, a substance similar to hydrogel, and placed at the RWM for local delivery. Local administration significantly increased the number of particles in the cochlea but formed a gradient of unevenly distributed nanoparticles with more nanoparticles residing in the cochlear basal turn. These studies established the feasibility of localized nanoparticle delivery across the round window (Tamura et al., 2005).

To increase the accumulation of nanoparticles in the inner ear, key modifications to the size, surface properties, and conjugated ligands can be made to affect diffusion kinetics and residence time of nanoparticles in the inner ear. Changing the size of PLGA nanoparticles to 150 and 300 nm enhanced entry into the inner ear. Functionalizing the surface of the nanoparticles with the non-ionic detergent pluronic acid 127 increased accumulation of particles in the inner ear after intracochlear delivery. Pluronic 127 increases the hydrophilicity of the nanoparticles to increase circulation time, reduce clearance, and enhance bioavailability (Zou et al., 2010b). Modifying particles with chitosan created a positively charged surface that increases the kinetics at which these particles enter the inner ear. Modifying nanoparticles with a hydrophilic surface using poloxamer 407 resulted in greater penetration and increased accumulation of particles in the perilymph. Lastly, tethering cell-penetrating peptide ligands to the surface, such as TAT peptides, could increase the transport of the nanoparticles into the inner ear through the round window. These studies demonstrate that facile surface functionalization can enhance nanoparticle delivery to the inner ear (Cai et al., 2017).

While PLGA nanoparticles can encapsulate drugs and allow delivery of a single drug, it can also be used to deliver multiple therapeutics for synergistic drug actions. To this end, the ability of PLGA nanoparticles to deliver a combination of three drugs, notoginsenoside R1, ginsenoside Rg1, and ginsenoside Rb1 to protect spiral ganglion neurons from cochlear ischemia was done. The total amount of R1, Rg1, and Rb1 in the perilymph increased 4. 0-, 3. 1-, and 7.1-fold, respectively, compared to the delivery of free drug solution. This demonstrated the ability of PLGA nanoparticles to simultaneously deliver multiple drugs to the inner ear for a potentially greater therapeutic effect (Cai et al., 2014).

For controlled delivery after injection, nanoparticles such as polymersomes can easily be modified, making them controllable by pH, magnetic fields and other factors (Meng et al., 2009). Polymersomes consisting of polyethylene glycol- polycaprolactone (PEG-PCL) diblock polymers encapsulate hydrophobic drugs into the center of the nanoparticles. Dye-labeled polymersomes delivered transtympanically or by cochleostomy showed a significant number of nanoparticles in the spiral ligament, the organ of Corti, and the spiral ganglion cells (Zhang et al., 2011a; Buckiová et al., 2012).

Amino acid and protein-based nanoparticles are biocompatible and increase drug retention time during delivery. Bovine serum albumin (BSA) proteins are biodegradable and can be used as a nanoparticle delivery system. Distribution of rhodamine-labeled BSA nanoparticles after intratympanic delivery increases the retention time of drugs in the middle ear. While BSA does not easily pass through the round window, it is thought to serve a similar purpose as hydrogels by increasing retention time at the round window and slowing degradation of drugs (Yu et al., 2014). Poly-amino acid-based nanoparticles such as poly (2-hydroxyethyl aspartamide) (PHEA) have been used for inner ear delivery. Dye encapsulated PHEA showed significant uptake in the organ of Corti, with dye accumulating in inner hair cells. PHEA nanoparticles may be advantageous for the delivery of therapeutics to the inner hair cells (Kim et al., 2015). While there is no known ligand on PHEA nanoparticles to target inner hair cells, preferential accumulation in inner hair cells may be attributed to their physical and chemical properties such as the surface charge and hydrophilicity of the particles and how they bind to the surface of inner hair cells before endocytosis of the nanoparticles.

Although size, surface properties, and surface functionalization can clearly affect polymeric nanoparticle retention time, degradation rate and uptake into cells, the mechanism by which each of these occurs will depend on nanoparticle design and the unique properties of different cell types. Structural, compositional, and functional interactions between nanoparticle and cells *in vivo* will likely constrain the efficiency of delivery and further studies are needed to fully elucidate the functional relationships between nanoparticles properties and their pharmacokinetic effects.

### Non-polymeric Nanoparticles

While polymeric nanoparticles are well studied for inner ear drug delivery, lipid-based, silica-based, and metallic nanoparticles have not been as well explored. Many of these non-polymeric nanoparticles are unique because of their magnetic, anti-microbial, tunable properties. Despite some of the useful qualities of non-polymeric nanoparticles, many are expensive, difficult to manufacture and only allow limited changes in surface properties.

Lipid-based nanoparticles can be used to deliver a range of therapeutics such as gadolinium to serve as an imaging contrasting agent (Zou et al., 2010a, 2012), doxorubicin for cancer treatment (Doxil) (Barenholz, 2012) and a transgene for gene therapy (Wareing et al., 1999). Lipid-based nanoparticles allow loading of large amounts of hydrophobic drugs, however, control over degradation and surface properties of the nanoparticle is limited. Fluorescently labeled lipid nanocapsules (LNCs) saturated in gelfoam and placed at the RWM can reach the spiral ganglion neuron cell bodies, nerve fibers, and spiral ligament fibrocytes within 30 min (Zou et al., 2008). LNCs did not cause hearing loss, cell death, or morphological changes in the inner ear, for up to 28 days suggesting low cytotoxicity (Zhang et al., 2011b).

Surface charge and hydrophilicity in lipid-based nanoparticles can change uptake and targeting. Addition of cationic and cationic-PEG (with a hydrophilic PEG chain) to phospholipid-based nanoparticle enhances entry into the RWM but showed a high level of cytotoxicity (Fröhlich, 2012). Using cationic-PEG particles to deliver dexamethasone to the mouse RWM provided a protective anti-inflammatory effect from kanamycin and furosemide treatment (Yang et al., 2018). Addition of cationic charge and increase of hydrophilicity at of phospholipid based nanoparticle can potentially be used for inner ear delivery if cytotoxicity is limited.

Lipid-based crystalline nanoparticles, called cubosomes formed from phytantriol lipids are of specific interest because they have a high drug loading capacity due to their large surface area. They are biodegradable and can incorporate hydrophobic, hydrophilic, and amphiphilic drugs into their matrix (Azmi et al., 2015). When nerve growth factor (NGF) was loaded into the phytantriol lipid-crystal nanoparticles (PHY-NGF) and delivered to the RWM, there was a significant 3.28 fold enhanced drug transport across the round window and a 4.8 fold increased maximum drug concentration compared to unloaded NGF treatment (Bu et al., 2015).

Metallic nanoparticles made of noble metals or iron oxide are used because of their bio-inert features, properties as MRI contrast imaging or ability for magnetofection. Fe_3_O_4_ nanoparticles oxidized by ceric ammonium nitrate (CAN) forms a CAN-Fe_2_O_3_ nanoparticle system that can cross both the round and oval window after delivery to the rat middle ear. The CAN-Fe_2_O_3_ accumulated in Reissner’s membrane, basilar membrane, and cochlear lateral wall. In the lateral wall, nanoparticles were found in the mesothelial cells of the scala tympani, the spiral ligament, and the stria vascularis. After 14 days all the nanoparticles were cleared from the inner ear (Zou et al., 2017). To improve tissue delivery of metallic nanoparticles, a magnetic field was used after intratympanic injection of prednisolone loaded magnetic nanoparticles in cisplatin-treated mice. Using a magnetic field to deliver drugs showed a significant reduction of hearing loss and increased outer hair cell survival from cisplatin-induced ototoxicity (Ramaswamy et al., 2017). Silver nanoparticles also have anti-microbial properties and serve as good contrasting agents for computed tomography (CT) heavy metal detection in the body. Distribution of silver nanoparticles assessed by micro-CT after transtympanic delivery was detected in the middle ear, ossicular chain, RWM, oval window, scala tympani, and Eustachian tube after 4 and 24 h. A distinct concentration gradient was found with fewer particles deeper into the inner ear. It was determined that entry to the inner ear was through the round and oval windows (Zou et al., 2015).

Lastly, silica-based nanoparticles are used because of their biocompatibility, biodegradability, and ease of surface modification and tuning. Distribution of Cy3 labeled silica nanoparticles in the inner ear after placement on the round window showed nanoparticle uptake in the inner hair cells, outer hair cells, spiral ganglion neurons, and supporting cells without any harmful effects on hearing (Praetorius et al., 2007). This demonstrated the safety and feasibility of using silica-based nanoparticles for inner ear drug delivery. Delivery of silica nanoparticle-based BDNF as neuroprotectant to deafened guinea pigs showed a significant improvement in SGN survival after noise-induced hearing loss (Wise et al., 2016). Similarly, hollow mesoporous silica nanoparticles with a zeolitic imidazolate shell can also be used for *in vitro* and *in vivo* delivery into inner ear cells (Xu et al., 2018).

The use of non-polymeric nanoparticles can greatly increase the variety of drugs for encapsulation and allow for a greater variety of physical and surface properties that are beneficial for developing drug delivery systems. While ease of modification and fine control of properties may not be as easy to control for non-polymeric nanoparticles, some of their innate properties such as magnetism, anti-microbial surface, and extremely high drug loading can make them attractive candidates for certain applications.

### Modified Nanoparticles for Cell-Specific Delivery

While nanoparticles themselves provide several advantages for increasing drug circulation time and preventing premature degradation of compounds, many drugs or therapeutics would ideally be delivered to a specific inner ear cell type for targeted therapies. Several different ligands have been used to enhance the cell-specific targeting of nanoparticles in the inner ear. Ligand-receptor mediated delivery allows the nanoparticles to bind to the cell surface of a specific cell type and have the nanoparticle endocytosed into the cell. Although the accumulation of nanoparticles into cells has been shown, the mechanism of uptake has not been rigorously tested. The rate and efficiency of uptake would depend on the endocytosis mechanisms in the cell type of interest.

Use of a human Nerve Growth Factor-derived peptide (hNGF_EE) conjugated to the surface of polymersomes drastically increased targeting to spiral ganglion neurons. Binding of the hNFG_EE peptide to the NGF receptor at the cell surface, presumably allows the nanoparticle to home into a cell, adhere to the cell and promote endocytosis of the nanoparticle into neurons. In addition to spiral ganglion neurons, Schwann cells, and nerve fibers can be targeted in organotypic cochlear cultures using the tyrosine kinase receptors and p75 neurotrophin receptors (Roy et al., 2010). Using polymersomes functionalized with a TET1 peptide allowed targeting to the cochlear nerve. The TET1 peptide binds to trisialoganglioside clostridial toxin receptor on nerve fibers to deliver nanoparticles (Zhang et al., 2012).

To target outer hair cells, PRESTIN binding peptides were identified using phage display and coupled to nanoparticles. PRESTIN is a protein expressed specifically in the lateral wall of the outer hair cells body. Two peptides A665 and A666 that bind to PRESTIN were conjugated to PEG-PCL polymersomes. Uptake of the polymersomes was tested in Chinese Hamster Ovary cells expressing PRESTIN and cochlear explants. Strong uptake of particles in the CHO cells and outer hair cells of rat cochleae was observed (Surovtseva et al., 2012). Using the A666 mediated PEG-PLA nanoparticle, dexamethasone was delivered to the round window prior to cisplatin treatment. A666 ligand conjugated particles were taken up by outer hair cells *in vivo* and promoted cell survival after ototoxic damage (Wang et al., 2018).

Cellular targeting using ligands is a relatively understudied field in inner ear drug delivery. The specificity of ligands for cell type-specific delivery is still lacking and display uptake of particles in unintended cell types. Lack of cellular specificity for delivery may limit the current use for some clinical applications such as cellular regeneration. The strong potential of targeting nanoparticles to specific cell types demonstrate the need for in-depth study of nanoparticle, ligand, and receptor interactions with cells found in the inner ear. While degradability, physical properties, and kinetics of nanoparticle systems have been well studied (Li et al., 2017), the target-specific delivery of nanoparticles an area that still needs to be developed to make a large impact in the field of inner ear therapeutics.

### Hybrid Hydrogel and Nanoparticle Systems

While nanoparticles and hydrogels each have advantages that significantly improve the efficacy and kinetics of delivery, combining the two materials allows synergistically increased control of drug delivery to the inner ear. Hydrogels provide prolonged exposure and increased residence time of loaded drugs, while nanoparticles decrease degradation and allow cell-specific delivery to the inner ear. One example of combining the two materials is the encapsulation of iron oxide nanoparticles into a Pluronic F127 hydrogel to create a “ferrogel.” Magnetic nanoparticles from the ferrogel cross the round window and are taken up by cells in the spiral ligament and Reissner’s membrane in human cadavers (Thaler et al., 2011). Application of a magnetic field could further enhance entry into the inner ear.

The combination of chitosan hydrogels and liposomes is another hybrid delivery platform. Chitosan hydrogels allowed sustained delivery, while liposomes improved the transport of nanoparticles through the round window. Tracking of dye-labeled particles after delivery showed the presence of nanoparticles in the perilymph and murine inner ear cells. Use of chitosan and liposome together increased the number of particles in the inner ear compared to nanoparticles alone (Lajud et al., 2015). This hybrid system was used in conjunction with PRESTIN binding peptides and the c-Jun N-terminal kinase (JNK) inhibitor, D-JNKi-1, to target outer hair cells. PRESTIN binding peptides promoted cell-specific delivery to outer hair cells while of D-JNKi-1 prevents apotosis. Together targeted delivery of D-JNKi-1 significantly protected hearing loss after noise-induced damage (Kayyali et al., 2018). Another example of a hybrid delivery system is the use of chitosan hydrogel with PLGA nanoparticles. Chitosan loaded with the drug interferon α-2 increased residence time of while PLGA prevented degradation. Use of both chitosan and PLGA increased the total amount of drug delivered to the perilymph (Dai et al., 2018).

The combination of film-forming agents and drug-loaded microspheres for intratympanic delivery to the round window are another example of a hybrid delivery system. While film-forming agents are not the same as hydrogels, they serve a similar purpose in slowing release kinetics of drug-loaded nanoparticles. These results showed that steady drug release can occur up to 30 days, and the particles in the film-forming agent remain localized on the round window for the same amount of time. This would not be possible with injection of a liquid suspension of nanoparticles. In addition, release kinetics could be tuned by modifying both the film-forming agent as well as the microspheres to allow greater control over delivery parameters depending on the desired application (Dormer et al., 2019). The combination of different materials such as hydrogels and nanoparticles can be used to modulate the pharmacokinetics of drug delivery to the inner ear via intratympanic injections.

### Conclusion and Future Perspectives

Microsurgical techniques for introducing therapeutics to the inner ear have shown that intratympanic and intracochlear injections are viable delivery methods. Although for most clinical applications, intratympanic delivery is more practical. When administering therapeutics via intratympanic injections, entry into the inner ear through the round or oval window depends on the molecular weight, charge, and other biophysical properties of the drug as it interacts with round or oval window membranes. The desired drug properties will depend on the inner ear disease to be treated. By tuning the properties of nanoparticles it may be possible to direct drugs to either the vestibular or auditory system by their preferential crossing through the round or oval window. Once inside the appropriate sensory organ, the residence time, binding and biological effect of the drug are affected by drainage, cellular fluid exchange and uptake by cells.

To optimize intratympanic therapeutic delivery, different tunable hydrogels allow for sustained release of drugs from the middle ear cavity and promote entry into the RWM before degradation. Nanoparticle platforms allow multiple drugs or therapeutics to be loaded, and prevent rapid degradation, increase residence time, and target appropriate cell types. By combining the tunable properties of hydrogels and nanoparticles, a versatile delivery platform with the desired properties can be engineered for a variety of drugs that cater to a specific clinical disease.

There still remains a large gap between preclinical and clinical research for employing and optimizing inner ear delivery systems. Studies on the safety and efficacy of biomaterials are still required. However, the promising results from preclinical models suggest that hydrogel and nanoparticle-based biomaterials are viable substrates for inner ear drug delivery based on their ability to pass through many of the physical and cellular barriers. As novel biomaterials are generated and a greater understanding of the inner ear physiology is achieved, biomaterial-based drug delivery to the inner ear will become more amenable for translational use.

## Author Contributions

CR wrote the nanomaterial part of the manuscript. Y-LY contributed to the description of the surgical procedures. S-TC generated the figures and edited the manuscript. K-BL edited the manuscript. KK contributed to the inner ear portion of the manuscript and edited the manuscript.

## Conflict of Interest

The authors declare that the research was conducted in the absence of any commercial or financial relationships that could be construed as a potential conflict of interest.
